# Autophagy regulates hepatocyte identity and epithelial-to-mesenchymal and mesenchymal-to-epithelial transitions promoting Snail degradation

**DOI:** 10.1038/cddis.2015.249

**Published:** 2015-09-10

**Authors:** G Grassi, G Di Caprio, L Santangelo, G M Fimia, A M Cozzolino, M Komatsu, G Ippolito, M Tripodi, T Alonzi

**Affiliations:** 1 National Institute for Infectious Diseases L. Spallanzani IRCCS, Rome, Italy; 2 Department of Cellular Biotechnologies and Hematology, Pasteur Institute-Cenci Bolognetti Foundation, Sapienza University of Rome, Rome, Italy; 3 Department of Biological and Environmental Sciences and Technologies (DiSTeBA), University of Salento, Lecce, Italy; 4 Department of Biochemistry, Niigata University Graduate School of Medical and Dental Sciences, Chuo-ku, Niigata, 951-8510, Japan

## Abstract

Epithelial-to-mesenchymal transition (EMT) and the reverse process mesenchymal-to-epithelial transition (MET) are events involved in development, wound healing and stem cell behaviour and contribute pathologically to cancer progression. The identification of the molecular mechanisms underlying these phenotypic conversions in hepatocytes are fundamental to design specific therapeutic strategies aimed at optimising liver repair. The role of autophagy in EMT/MET processes of hepatocytes was investigated in liver-specific autophagy-deficient mice (Alb-Cre;ATG7^fl/fl^) and using the nontumorigenic immortalised hepatocytes cell line MMH. Autophagy deficiency *in vivo* reduces epithelial markers' expression and increases the levels of mesenchymal markers. These alterations are associated with an increased protein level of the EMT master regulator Snail, without transcriptional induction. Interestingly, we found that autophagy degrades Snail in a p62/SQSTM1 (Sequestosome-1)-dependent manner. Moreover, accordingly to a pro-epithelial function, we observed that autophagy stimulation strongly affects EMT progression, whereas it is necessary for MET. Finally, we found that the EMT induced by TGF*β* affects the autophagy flux, indicating that these processes regulate each other. Overall, we found that autophagy regulates the phenotype plasticity of hepatocytes promoting their epithelial identity through the inhibition of the mesenchymal programme.

The epithelial-to-mesenchymal transition (EMT) is a complex phenomenon by which epithelial-polarised cells lose their polarity and cell–cell connections, acquiring mesenchymal characteristics of motility and invasiveness. During EMT, epithelial cells change their cytoskeleton and signalling pathways, which reorganise cell shape and gene expression, orchestrated by a restricted number of master transcription factors, and among these Snail and Slug have a main role.^1^ The reverse process mesenchymal-to-epithelial transition (MET), much less characterised at the molecular level, restores the specific epithelial identity.^2^

EMT and MET are well-established biological events occurring sequentially in development and organogenesis, which are reactivated and regulated in wound healing, tissue repair, fibrosis and cancer progression, in response to signals from the proximal microenvironment. Therefore, the identification of the molecular mechanisms underlying these phenotypic conversions in hepatocytes are fundamental to understand the pathogenesis of liver diseases.

Autophagy is an intracellular pathway by which lysosomes degrade and recycle proteins and cellular organelles. The processes activated for lysosome delivery have defined three types of autophagy: macroautophagy, microautophagy, and chaperone-mediated autophagy. During macroautophagy (herein after referred as autophagy), the materials to be degraded are delivered to the lysosome through *de novo* generation of a double-membrane vesicles, termed autophagosomes, that sequester cytosolic targets and then fuse with the lysosomes. Autophagic processes can be either constitutive or activated in response to different stimuli.^3^

In the liver, autophagy has different functions important for the organ homeostasis either in physiological conditions, contributing to the maintenance of the energetic balance, cellular quality control and the regulation of proteins turnover, or in response to pathological stimuli, such as viral and bacterial infections, DNA damage, toxic injuries or nutrient deprivation. Autophagy dysregulation is involved in the pathogenesis of different liver diseases, such as metabolic disorders, protein conformational diseases, viral infections and hepatocarcinogenesis.^4^

In animal models, hepatocyte-specific autophagy deficiency cause liver injury, severe hepatomegaly and tumorigenesis. Autophagy-deficient hepatocytes accumulate protein aggregates together with the sequestosome 1 (SQSTM1 or p62 and hereinafter referred to as p62), an autophagy substrate and cargo protein normally incorporated and degraded into the autophagosomes in association with proteins that have to be eliminated.^5, 6^ In particular, the accumulation of p62 leads to the activation of Keap1 (Kelch-like ECH-associated protein 1)–Nrf2 (nuclear factor (erythroid-derived 2)-like 2) pathway, one of the major regulator of cytoprotective responses to oxidative and electrophilic stress. p62 activates the transcription factor Nrf2 by binding and inhibiting Keap1, an adaptor of the ubiquitin ligase complex that targets Nrf2 for degradation.^7, 8^ Although observations from Nrf2- or p62-deficient mice suggest that persistent activation of Nrf2, caused by the impaired turnover of p62, accounts for most of the pathogenic effects in the liver,^9, 10^ little information on the impact of autophagy in hepatocytes differentiation is available.

In this work, we investigated the functional relationship between autophagy and hepatocyte differentiation with a particular focus on EMT/MET dynamics. The role of autophagy in the balance between epithelial and mesenchymal states was investigated both *in vivo*, using liver-specific ATG7-deficient mice (Alb-Cre;ATG7^fl/fl^),^6^ and *in vitro*, using the immortalised nontumorigenic hepatocytic cell line MMH (Met murine hepatocytes), which undergoes EMT upon transforming growth factor beta1 (herein referred as TGF*β*) treatment and to MET upon TGF*β* withdrawal.^11, 12, 13, 14, 15^ Here we report that lack of autophagy leads to a decreased expression of epithelial markers with the concomitant induction of the mesenchymal programme. Autophagy promotes the degradation of Snail in hepatocytes through the action of p62. Moreover, while the lack of autophagy leads to an increased EMT and an impaired MET, autophagy induction prevents the transition of hepatocytes from the epithelial to mesenchymal state.

Overall, our data unveil a new role played by autophagy in hepatocyte pathophysiology, demonstrating that it contributes to the maintenance of hepatocyte differentiation and epithelial cell identity.

## Results

### Autophagy deficiency in hepatocytes correlates with alteration of epithelial and mesenchymal markers' levels

**Figure 1:**
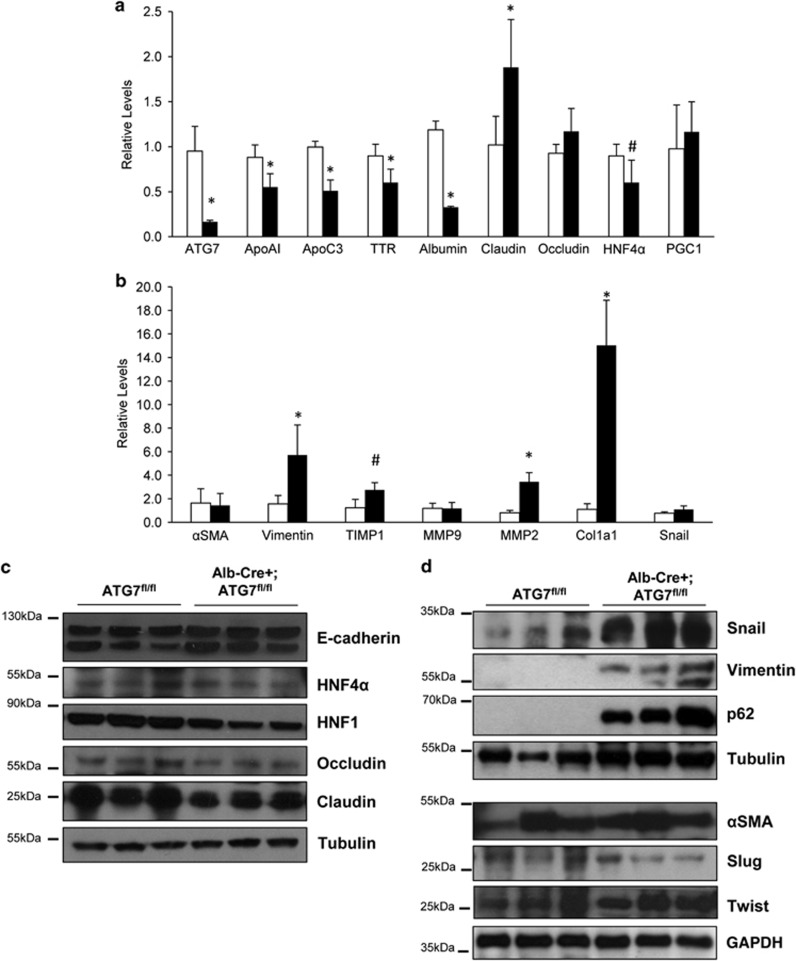
Liver-specific autophagy-deficient mice express decreased levels of epithelial genes and increased mesenchymal markers levels. (**a** and **b**) mRNA levels of the indicated genes were measured by quantitative real-time PCR (qPCR) in liver extracts of mice either autophagy-proficient (ATG7^fl/fl^; white column; *n*=8) or autophagy-deficient (Alb-Cre+ATG7^fl/fl^; black column; *n*=9). The values calculated by ΔΔCT method are relative to L34 mRNA levels and expressed as fold of change with respect to control mice. Data are expressed as mean±S.D. *P<0.01; #*P*<0.05; *P*-values were calculated by Mann–Whitney *U*-test. (**c** and **d**) Immunoblotting analysis for epithelial (**c**) and mesenchymal (**d**) markers of whole liver extracts of mice either autophagy-proficient (ATG7^fl/fl^) or autophagy-deficient (Alb-Cre+ATG7^fl/fl^). Tubulin and GAPDH were used for protein loading control

In order to investigate whether autophagy impairment influences the hepatic gene expression, we measured the levels of several epithelial and mesenchymal mRNAs in ATG7 liver-specific deficient mice (Alb-Cre;ATG7^fl/fl^) and their autophagy-sufficient wild type littermates (ATG7^fl/fl^).^6^ As shown in Figure 1a, the Alb-Cre;ATG7^fl/fl^ mice display a general reduced expression of epithelial-specific genes (i.e., apolipoprotein(apo)A-I, apoC3, transthyretin, Albumin and Hepatocyte nuclear factor (HNF) 4 alpha (HNF4*α*). On the other hand, increased levels of several mesenchymal-specific genes (i.e., Vimentin, matrix metalloproteinase (MMP)-2, collagen type I alpha 1, tissue inhibitor of metalloproteinase 1) were found in the autophagy-deficient livers (Figure 1b). Western blotting analysis of the whole liver extracts of these mice confirmed the downregulation of some epithelial markers such as HNF1, HNF4*α*, Occludin and Claudin (Figure 1c) and the upregulation of several mesenchymal markers such as Vimentin, alpha smooth muscle actin (*α*SMA), as well as the EMT-related transcriptional factors Snail, Slug and Twist (Figure 1d). The levels of p62 were also found increased, as expected by the lack of autophagy (Figure 1d).^6^ These results suggest that in the autophagy-deficient livers the fine regulation of the balance between the mesenchymal and epithelial programmes is altered.

**Figure 2:**
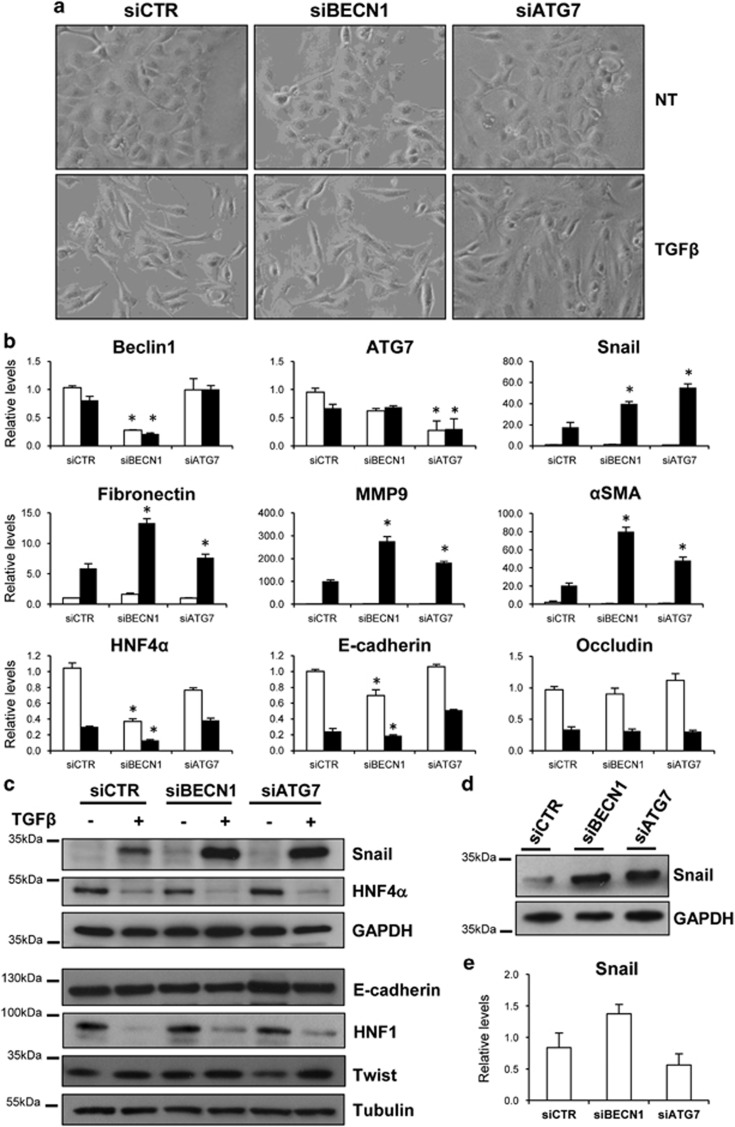
Lack of autophagy increases the mesenchymal markers expression. (**a**) Phase-contrast images of autophagy-deficient (siBECN1 or siATG7) or autophagy-proficient (siCTR) cells either untreated (NT) or treated with TGF*β* (2 ng/ml) for 24 h. (**b**) mRNAs levels of the indicated genes were measured by qPCR in siCTR, siBECN1 and siATG7 cells either untreated (white columns) or treated with TGF*β* (2 ng/ml) for 24 h (black columns). The values calculated by ΔΔCT method are relative to L34 mRNA levels and expressed as fold of change with respect to untreated siCTR cells. Data are shown as mean±S.D. of three independent experiments. *P<0.05; *P*-values were calculated by Mann–Whitney *U*-test. (**c** and **d**) The protein levels of epithelial and mesenchymal markers were measured in autophagy-proficient (siCTR) or autophagy-deficient (siBECN1; siATG7) cells either untreated or treated with TGF*β* (2 ng/ml) for 24 h by western blotting. GAPDH (glyceraldehyde 3-phosphate dehydrogenase) was used for protein loading control. In panel (**d**), a higher amount of whole cell extracts proteins (30 *versus* 10 *μ*g in panel (**c**)) were loaded to obtain a better Snail signal. (**e**) mRNA of Snail were measured as in panel (**b**) in untreated siCTR, siBECN1 and siATG7 cells. *P<0.05 *versus* siCTR

To determine a possible molecular link between the autophagy process and this phenotypic regulation, we used a well-characterised cellular model, the differentiated, immortalised, nontumorigenic hepatocytic cell line MMH, capable of undergoing a reversible EMT upon TGF*β* stimulation.^12, 13, 14, 15^ First, it was tested whether the lack of autophagy influences the TGF*β*-dependent EMT. To this aim, the expression of Beclin1 (BECN1) and ATG7, two fundamental genes for autophagy induction, was silenced by specific siRNAs in hepatocytes. As shown in Figure 2, while the lack of either BECN1 or ATG7 did not affect significantly the TGF*β*-induced morphological transition into fibroblastoid phenotype (Figure 2a), it resulted in increased expression levels of mesenchymal-specific genes such as MMP9, *α*SMA, Fibronectin and Snail, when compared with cells transfected with the negative control siRNA (Figure 2b). Although the protein levels of E-cadherin, HNF1, HNF4*α* and Twist were not significantly affected, Snail protein was significantly higher in autophagy-deficient cells 24 h after TGF*β* treatment (Figure 2c), thus further suggesting that lack of autophagy facilitates the transition from epithelial to mesenchymal state.

More interestingly, the silencing of either BECN1 or ATG7 induced increased levels of Snail protein also in untreated cells (Figure 2d), without a significant upregulation of its gene expression (Figure 2e). This result suggests that in hepatocytes Snail protein is constitutively degraded by autophagy.

### Autophagy degrades Snail through p62

In order to identify the molecular mechanism responsible for Snail accumulation in the absence of autophagy, we first analysed whether Snail interacts with the autophagy machinery.

**Figure 3:**
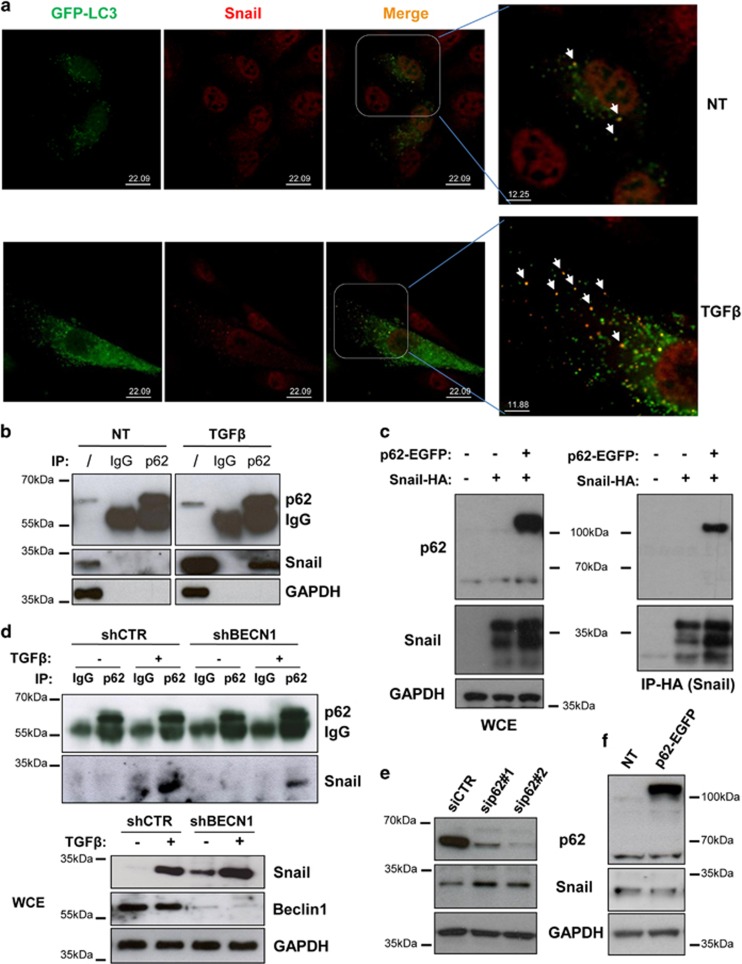
Autophagy degrades Snail in hepatocytes through the action of p62. (**a**) Colocalisation of LC3 with Snail was analysed by immunofluorescence staining for Snail (red) in hepatocytes expressing GFP-LC3 (green). Cells, either untreated or stimulated with TGF*β*, were treated with BafA1 (5 ng/ml) for 3 h, fixed, permeabilised, stained with an anti-Snail antibody and analysed by confocal microscopy. In the magnified panels, arrows indicate the colocalisation of GFP-LC3 with Snail. (**b**) Immunoblotting analysis for Snail and p62 following immunoprecipitation of either p62 or using an isotype-matched control mAb from lysates of parental cells either untreated or stimulated with TGF*β* (2 ng/ml) for 3 h. GAPDH (glyceraldehyde 3-phosphate dehydrogenase) was used for protein loading control. (**c**) Immunoblotting analysis for Snail and p62 following immunoprecipitation for HA (Snail) in parental cells or in hepatocytes overexpressing Snail-HA either in the presence or absence of p62-EGFP (48 h posttransfection). GAPDH was used for protein loading control. (**d**) Immunoblotting analysis of Snail and p62 following immunoprecipitation of either p62 or using an isotype-matched control mAb from lysates of autophagy-deficient (shBECN1) or control (shCTR) cells either untreated or stimulated with TGF*β* (2 ng/ml) for 3 h. In whole-cell extracts (bottom panels), BECN1 and GAPDH were used for analysing the BECN1-silencing levels and as protein loading control, respectively. (**e** and **f**) Immunoblotting analysis for Snail and p62 of hepatocytes in which the expression of p62 was either inhibited by two specific siRNAs (**e**) or increased by transfecting a p62-EGFP-expressing vector (**f**). GAPDH levels were used for protein loading control

First, we monitored the localisation of the microtubule-associated protein 1A/1B-light chain 3 (LC3), a protein necessary for autophagosome formation, and Snail. As showed by confocal analysis of hepatocytes stably expressing the green fluorescent protein-tagged LC3 (GFP-LC3), Snail colocalised with autophagosomes (i.e., GFP-LC3 puncta), in either untreated or TGF*β*-treated (i.e., epithelial or mesenchymal phenotypes, respectively) cells (Figure 3a), thus supporting the hypothesis that autophagy degrades the EMT master regulator Snail in hepatocytes.

In the attempt to identify the cargo protein responsible for Snail degradation, we focussed our attention on p62, which is a regulator and substrate of autophagy and serves as a receptor for selective autophagic clearance of protein aggregates and organelles.^16^

First, we analysed whether p62 interacts with Snail using co-immunoprecipitation experiments. As shown in Figure 3b, p62 co-immunoprecipitated with Snail specifically in TGF*β*-treated cells. However, the very low levels of Snail protein in untreated cells could explain the lack of interaction with p62. In fact, when the human Snail-HA (hematoagglutinin) and p62-EGFP (enhanced GFP) were overexpressed in hepatocytes, they co-immunoprecipitated in the absence of treatment, therefore demonstrating that TGF*β* is not necessary for Snail-p62 interaction (Figure 3c).

Next, we tested whether a functional autophagy is required for p62-mediated Snail degradation using autophagy-deficient hepatocyte cells in which BECN1 was stably silenced by a short hairpin RNA (shRNA) approach (shBECN1). These cells expressed higher levels of Snail protein, as evidenced by both western blotting analysis (Figure 3d; lower panel) and immunofluorescence (Supplementary Figure S1), thus confirming the results obtained with the siRNA approach (Figure 2d). Co-immunoprecipitation experiments evidenced a lower level of p62–Snail interaction in TGF*β*-treated shBECN1 cells when compared with the control-silenced cells (shCTR) (Figure 3d). Although the immunoprecipitation assay is not quantitative, this result suggests that an autophagy-sufficient environment promotes the p62–Snail interaction. To establish whether p62 either actively shuttles Snail to the autophagosomes for degradation or mediates its accumulation by inhibiting a negative regulator, similarly to what reported for Twist or Nrf2,^6, 17, 18^ p62 was silenced by siRNAs. As shown in Figure 3e, the lack of p62 resulted in increased levels of Snail, thus strongly indicating that p62 acts as an active cargo for the autophagy-mediated degradation of Snail. This result was corroborated by the lack of increased Snail protein level in p62-overexpressing cells (Figure 3f).

Overall, these data demonstrate that autophagy degrades Snail through a p62-mediated mechanism in hepatocytes.

### TGF*β*-induced EMT causes autophagy impairment

**Figure 4:**
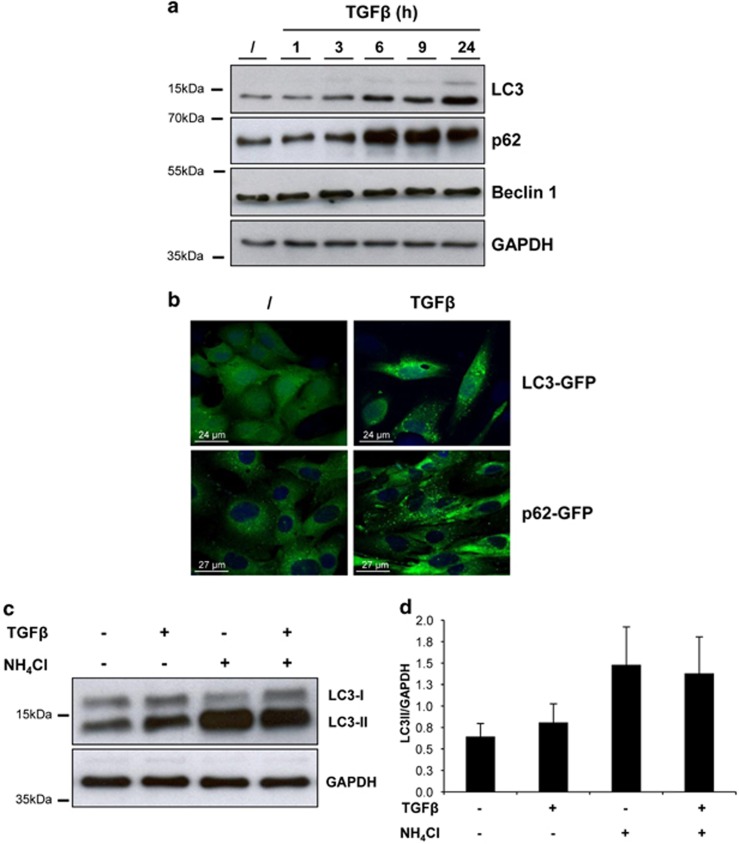
TGF*β* induces impaired autophagic flux in nontumorigenic hepatocytes. (**a**) Immunoblotting analysis of LC3, p62 and BECN1 in hepatocytes treated with TFG*β* (2 ng/ml) for the indicated hours. GAPDH (glyceraldehyde 3-phosphate dehydrogenase) levels were used as loading control. (**b**) Fluorescence analysis of the localisation of LC3 or p62 in hepatocytes expressing either LC3-GFP or p62-GFP, respectively, following treatment with TGF*β* (2 ng/ml) for 24 h. The blue is a ToPRO3 nuclear counterstaining. (**c**) Immunoblotting analysis of LC3 in hepatocytes treated with TFG*β* (2 ng/ml; 9 h) either in the presence or absence of the autophagy inhibitor NH_4_Cl (20 mM) for the last hour of treatment. GAPDH was used for protein loading control. A representative experiment out of four is shown. (**d**) Quantification of the ratio of LC3-II to GAPDH band intensities obtained by immunoblotting analysis as in panel (**c**), expressed as average ±S.D. (*n*=4)

In the light of the evidence that Snail protein is a substrate of autophagy, we decided to investigate whether autophagy is modulated during EMT by analysing protein levels and cellular localisation of LC3 and p62. First, we monitored the electrophoretic mobility of LC3, which is known to change upon autophagy induction, as the cytosolic form of LC3 (LC3-I) is covalently conjugated to phosphatidylethanolamine generating the autophagosomal membrane-bound form LC3-II. As shown in Figure 4a, TGF*β* significantly increased the endogenous LC3-I to LC3-II conversion starting 3 h after treatment. Moreover, TGF*β* induced the LC3 dots' formation, which indicates autophagosome generation, as revealed by cells stably expressing GFP-LC3 (Figure 4b, top panels).

Next, the levels of p62 were analysed. p62 serves as a readout of autophagy degradation as it is known to bind to ubiquitinated proteins for their delivery to autophagosomes and degradation.^19^ In TGF*β*-treated hepatocytes, we observed an accumulation of p62 starting from 3 to 6 h posttreatment (Figure 4a). Moreover, 24 h post-TGF*β* treatment p62 was mainly found with a dot distribution, which suggests an autophagosomal localisation (Figure 4b, bottom panels). These results indicate that the TGF*β* inhibits the final step of the autophagic flux.

To better elucidate whether the autophagy is inhibited during EMT, we analysed the autophagy flux by measuring LC3-II levels in cells treated with TGF*β*, either in the absence or presence of the lysosome-neutralising agent NH_4_Cl, which prevents the degradation of autophagosomes. As shown in Figures 4c and d, while NH_4_Cl causes an accumulation of LC3-II in untreated cells, co-treatment with TFG*β* does not increase LC3-II levels, thus demonstrating that TGF*β* inhibits the degradation of LC3 rather than increasing the autophagy flux.

Overall, these results suggest that the autophagy activity is impaired during the TGF*β*-induced EMT.

### Induction of autophagy inhibits the TGF*β*-mediated EMT

**Figure 5:**
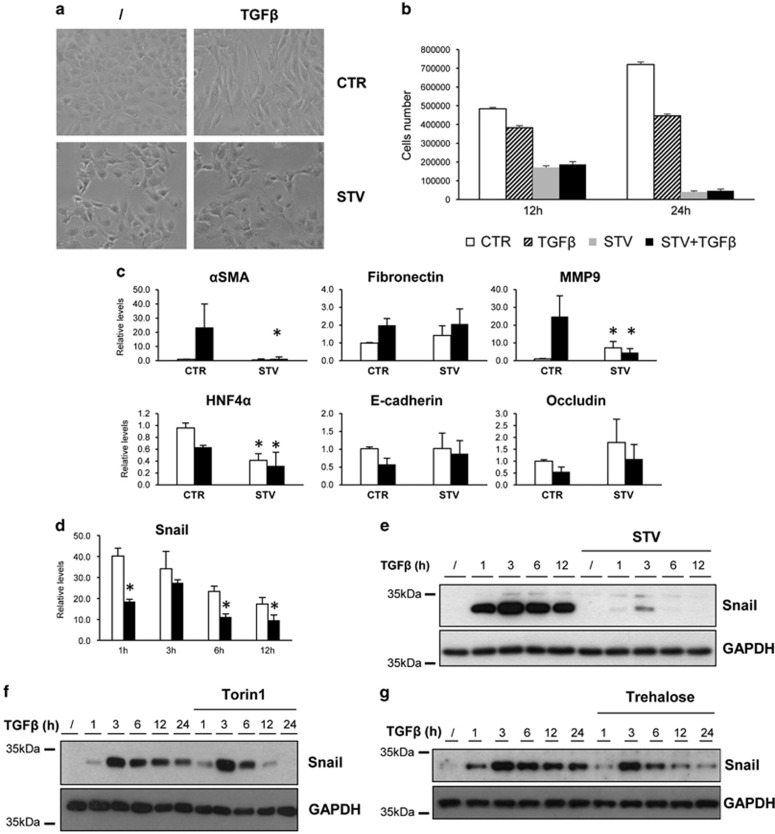
Autophagy stimulation inhibits the TGF*β*-induced EMT and promotes Snail degradation in hepatocytes. (**a**) Phase-contrast images of cells treated with TGF*β* (2 ng/ml) in the absence (CTR) or presence (STV) of the starvation medium for 12 h. (**b**) Cell counts of hepatocytes cultivated either in normal medium alone (CTR) or with 2 ng/ml of TGF*β* (TGF*β*) or cultivated in the starvation medium alone (STV) or with 2 ng/ml of TGF*β* (STV+TGF*β*) for 12 or 24 h, as indicated. (**c**) Levels of mRNAs of the indicated genes were measured by qPCR in CTR or STV cells either untreated (white columns) or treated with TGF*β* (2 ng/ml) (black columns) for 12 h. (**d**) Levels of Snail mRNA were measured by qPCR in CTR (white columns) or STV (black columns) cells treated with TGF*β* (2 ng/ml) for different time periods as indicated. The values calculated by ΔΔCT method are relative to L34 mRNA levels and expressed as fold of change with respect to untreated cells. Data are expressed as mean±S.D. of three independent experiments. *P<0,05; *P*-values were calculated by Mann–Whitney *U*-test. (**e**–**g**) Immunoblotting analysis for Snail in hepatocytes treated with TFG*β* (2ng/ml) for different time periods as indicated. One hour before TFG*β* treatment, cells were left untreated or co-treated with starvation medium (STV) (**e**), Torin1 (1 *μ*M) (**f**) or Trehalose (100 mM) (**g**). GAPDH (glyceraldehyde 3-phosphate dehydrogenase) was used as protein loading control

As TGF*β* impairs autophagy, we asked whether stimulation of autophagy could influence EMT. As shown in Figure 5, nutrient deprivation (starvation) significantly inhibited the TGF*β*-mediated EMT: (i) the fibroblastoid morphology was prevented (Figure 5a); (ii) the repression of epithelial genes was significantly affected; and (iii) the induction of mesenchymal genes transcription was impaired (Figure 5c). To test whether the TGF*β* pathway is functional in starved hepatocytes, we analysed the expression levels of Snail, which is a TGF*β* immediate early gene. Upon starvation, TGF*β* was able to stimulate Snail gene transcription (Figure 5d), thus indicating that the TGF*β* pathway was not inhibited by nutrient deprivation. However, despite the mRNA production, protein levels of Snail were significantly decreased at each time point analysed (Figure 5e), therefore confirming that autophagy affects Snail production by a posttranslational mechanism.

It is worth to note that the TGF*β*-induced EMT was analysed up to 12 h posttreatment, when the EMT programme is induced while not yet complete. This was the last time point studied, because prolonged nutrient deprivation and TGF*β* treatment caused massive cell death (Figure 5b).

To further assess the inhibitory role of autophagy in the TGF*β*-mediated EMT, we extended our observation to other autophagy inducers, the mTOR inhibitor Torin1^20^ and Trehalose, an autophagy inducer acting via a not yet identified mTOR-independent pathway.^21^ In both Torin1- and Trehalose-treated cells, EMT was significantly affected (Supplementary Figures S2 and S3, respectively), and the levels of Snail protein decreased (Figures 5f and g, respectively), despite its mRNA levels was almost comparable to those of TGF*β*-teated cells (Supplementary Figures S2D and S3D, respectively).

Overall, these data indicated that autophagy counteracts the EMT process triggered by TGF*β*.

### Lack of autophagy affects the hepatocytes MET

**Figure 6:**
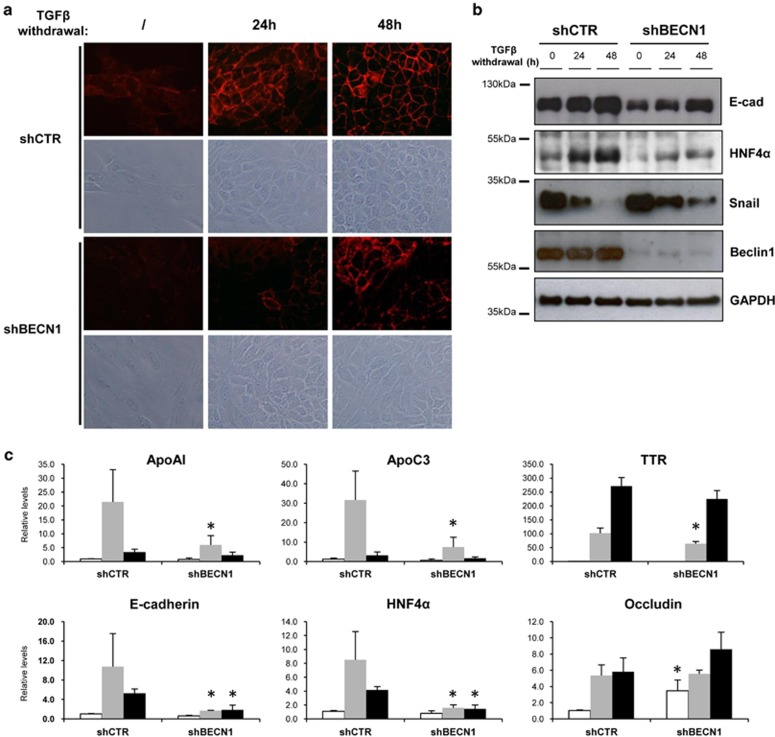
Lack of autophagy impairs the MET in hepatocytes. (**a**) Phase-contrast images and immunofluorescence analysis of the localisation of E-cadherin in hepatocytes. Cells were treated with TGF*β* (2ng/ml) for 24 h, extensively washed and after 0, 24 or 48 h of culture in the absence of TGF*β* cells were fixed and stained for E-cadherin. (**b**) Immunoblotting analysis of E-cadherin, HNF4*α*, Snail and BECN1 in shCTR or shBECN1 hepatocytes. Cells were lysed after 24 h of TGF*β* (2 ng/ml) treatment or after 24 or 48 h of further culture in the absence of TGF*β*. GAPDH (glyceraldehyde 3-phosphate dehydrogenase) was used as a loading control. (**c**) mRNA levels of the indicated genes were measured by qPCR in shCTR or shBECN1 hepatocytes during EMT (24 h of 2 ng/ml of TGF*β*; white columns) or during MET (further cultured in the absence of TGF*β* for 24 (grey columns) or 48 (black columns) hours). The values calculated by ΔΔCT method are relative to L34 mRNA levels. Data are expressed as mean±S.D. of three independent experiments. *P<0.05; *P*-values were calculated by Mann–Whitney *U*-test

As the inhibition of autophagy resulted in the upregulation of the mesenchymal programme, we asked whether autophagy contributes also to MET, the reacquisition of the epithelial phenotype upon mesenchymal transition. To address this issue, EMT was first induced in either shCTR or shBECN1 cells by 24 h of TGF*β* treatment, and after TGF*β* withdrawal, MET was analysed for the following 48 h. As shown in Figure 6, in autophagy-inhibited cells the MET process was significantly delayed compared with that of shCTR cells as assessed by: (i) the delayed restoration of epithelial morphology and E-cadherin localisation (Figure 6a), (ii) the decreased protein levels of E-cadherin, HNF4*α* (Figure 6b), (iii) the increased levels of Snail (Figure 6b), and (iv) the reduced expression of epithelial genes (i.e., HNF4α, E-cadherin, ApoC3 and ApoA-I) (Figure 6c).

These results demonstrated that autophagy is required for the restoration of hepatocyte differentiation following an environmental stress, such as the EMT-inducer TGF*β*.

## Discussion

Autophagy is fundamental in hepatic homeostasis, for both basic functions (i.e., energetic balance, removal of misfolded proteins and damaged organelles) and the response to liver insults (i.e., niche milieu modifications, pathogens infections, DNA damage, toxic stimuli).^4, 22^ The main contribution of this work is the identification of a new role for autophagy in both conditions: the maintenance of a stable differentiation state of hepatocytes and in the EMT/MET processes.

Here we reported that autophagy actively degrades the EMT master regulator Snail under basal condition, both *in vivo* and *in vitro*, and supports the reacquisition of the epithelial identity during MET. Notably, our data proved that autophagy inhibition occurs during induction of EMT.

The constitutive degradation of Snail protein is important for epithelial identity. In fact, it is becoming evident that the balance between mesenchymal and epithelial master regulators (i.e., Snail and HNF4*α* and HNF1*α*, respectively), integrated by specific microRNAs regulation, is responsible for stable maintenance of hepatocyte identity, as well as for the EMT/MET dynamics.^2^

Snail is a highly unstable protein, with a half-life of about 25 min, and its degradation has been described to be triggered by glycogen synthase kinase 3*β* phosphorylation and mediated by the ubiquitin proteasome system (UPS).^23^ In autophagy-competent cells, Snail is degraded by both autophagy and proteasome (Supplementary Figure S4). The raise in Snail levels that we observed in autophagy-deficient hepatocytes was due to a decreased Snail degradation mediated by p62, a multifunctional protein implicated in several signal transduction pathways and in regulating selective autophagy.^16^ Increased Snail levels were found in p62-silenced cells (Figure 3e) and not in the p62-overexpressing cells (Figure 3f), thus indicating that, in hepatocytes, p62 does not increase Snail stabilisation through the inhibition of its UPS-mediated degradation, similarly to what found for the transcription factors Nrf2^7, 8^ or Twist.^17, 18^ As, in addition to hepatocytes, we found increased Snail levels in autophagy-deficient ambra1-null mouse embryonic fibroblasts^24^ (Supplementary Figure S5) and that the activation of autophagy degrades Snail in human breast cancer and in glioblastoma cells,^25, 26^ we could speculate that autophagy is a general mechanism controlling Snail levels, alternative and synergistic with UPS. In addition to Snail protein degradation, we found that autophagy could also affect the Snail transcriptional regulation induced by TGF*β*; in fact, in the absence of autophagy (i.e., siATG7 and siBECN1) Snail induction was increased (Figure 2b) while autophagy activation (i.e., starvation, mTOR inhibition or trehalose stimulation) lead to a lower level of Snail mRNA (Figure 5d, Supplementary Figures S2D and S3D), thus indicating that activators and/or repressor of Snail gene are modulated by autophagy. This point should be further investigated.

Although Snail has been described to be important for cell cycle control and for mesenchymal identity in hepatocytes,^12, 27, 28^ it has to be noted that in basal condition autophagy inhibition *per se* is not sufficient to trigger a 'spontaneous' EMT or a cell cycle inhibition. However, as Snail is sufficient to repress a broad repertoire of epithelial genes, including epithelial master regulator HNF4*α*, it could be hypothesised that the impairment of its degradation renders hepatocytes prone to EMT.

A second hint of interest of our data concerns the inhibitory role of TGF*β* on the autophagic flux. Although the molecular mechanism was not investigated, we ruled out a transcriptional downregulation of autophagy genes (i.e., ATG5, ATG7, BECN1, ATG7 and p62) induced by TGF*β* (data not shown). The inhibitory effect of TGF*β* on autophagy should not be a surprise as it is known that it activates the autophagy inhibitor mTOR through the PI3K–AKT pathway.^29^ However, it has been reported that in hepatoma cells TGF*β* activates autophagy.^30^ Interestingly, this opposite effect of TGF*β* on autophagy in tumorigenic *versus* nontumorigenic hepatocytes recalls the well-known 'TGF*β* paradox in cancer progression', where cell growth is inhibited in early stage and promoted at late stage of tumors.^31, 32^ As the 'cancer paradox' is also true for autophagy, which has either antitumoral or pro-tumoral functions,^33^ it is tempting to speculate that the TGF*β*-mediated dual activity on cancer progression could be exerted also through a different regulation of autophagy. In agreement with this hypothesis, while our results suggested that the lack of autophagy increases the mesenchymal state of nontumorigenic hepatocytes, the work of Peng *et al.*^34^ showed that in hepatocellular carcinoma cells autophagy activates EMT, promoting their invasion potential. This hypothesis is also indirectly supported by results obtained on either normal epithelial mammary gland cells in which autophagy activation decreases EMT and cell migration^35, 36^ or immortilised podocytes in which autophagy inhibition leads to increased mesenchymal and decreased epithelial markers.^37^

Our results also demonstrated that autophagy is required for a successful MET. It could be speculated that autophagy is necessary for the restoration of hepatocytes differentiation and functions after an organ insult. Although we showed that liver-specific Atg7 knockout mice display a decreased epithelial-specific and increased mesenchymal-specific gene expression, thus supporting this hypothesis, we cannot rule out that this altered gene regulation could be due to a secondary effect generated by the liver injury developed by these mice. However, it is evident that the failure to properly execute the autophagy programme renders hepatocytes vulnerable to stressors and unable to provide the high energetic demands, which are necessary to restore the normal organ functionality. Besides the results here described on Alb-Cre+ATG7^fl/fl^ mice and MMH cells, there are evidences supporting this assumption. Autophagy deficiency in the liver contributes to parenchymal damage (i) during a normal diet, which is exacerbated after a methionine choline-deficient diet,^38^ and (ii) in response to a tumor necrosis factor-dependent liver injury.^39^ It has been also reported that autophagy helps the hepatocyte polarisation during the bile canaliculi formation, when cultured in a collagen sandwich system.^40^ Moreover, in the light of our results, it is a very interesting observation that the expression of Snail in hepatocytes has a pro-fibrogenic role, in fact hepatocyte-specific Snail deficiency (Alb-Cre+Snail^fl/fl^ mice) reduced liver fibrosis induced by the carbon tetrachloride treatment.^41^ It is, therefore, tempting to speculate that the autophagy-mediated Snail degradation is helpful for contrasting fibrogenesis. According to this hypothesis, it has been reported that autophagy in hepatocytes exerts an antifibrogenic effect.^10, 42, 43, 44^ Interestingly, during liver fibrosis autophagy exerts a functional paradox, it has an antifibrogenic effect in hepatocytes or in macrophages while it has a pro-fibrogenic role in hepatic stellate cells.^45^ These opposite effects on fibrosis highlight the importance of understanding the role played by autophagy in liver pathophysiology and to influence the choice for a cell-specific pro-autophagy or antiautophagy therapeutic approach.

Overall, our results showed that autophagy contributes to the maintenance of a stable differentiation state of hepatocytes, counteracting the mesenchymal program either at the basal level or during the phenotype transitions between mesenchymal and epithelial state.

## Materials and Methods

### Mouse liver samples, cells and culture conditions

RNA and whole-liver protein extracts of 4-, 9- and 11-month-old autophagy-deficient (Alb-Cre;ATG7^fl/fl^) or autophagy-sufficient mice (ATG7^fl/fl^) were isolated as already described.^5, 6^

The nontumorigenic differentiated epithelial MMH cell line was already described^11^ (see Supplementary Materials and Methods section for more details). Cells were grown on collagen type I (BD Biosciences, San Jose, CA, USA) coated Petri dishes in complete medium RPMI 1640 (Sigma-Aldrich, St Louis, MO, USA) supplemented with 10% FBS (Gibco, Life Technologies, Grand Island, NY, USA), 50 ng/ml EGF (PeproTech, Inc., Rocky Hill, NJ, USA), 30 ng/ml IGF-II (PeproTech), 10 mg/ml insulin (Roche, Mannheim, Germany), 2 mM  L-glutamine and 1% penicillin/streptomycin solution (Sigma-Aldrich). For EMT induction, cells were treated with 2 ng/ml of TGF*β* (PeproTech). For MET induction, cells were initially transdifferentiated into mesenchymal cells by 24 h of TGF*β* (2 ng/ml) extensively washed and cultivated in complete medium for 48 h.

For autophagy induction, cells were treated 1 h before TGF*β* treatment with: (i) Earle's balanced salt solution (Sigma-Aldrich) for nutrient deprivation (Starvation); (ii) Torin 1 (1 *μ*M) (Tocris Bioscience Bristol, UK) or (iii) Trehalose (100 mM) (Sigma-Aldrich).

For autophagy inhibition cells were either transfected with specific siRNAs targeting BECN1 or ATG7 or infected with retroviruses expressing a shRNA specific for BECN1 (see Supplementary Materials and Methods section for details).

To block lysosomal activity, cells were incubated in complete medium in the presence of NH_4_Cl (20 mM; Sigma-Aldrich).

For autophagy markers' localisation, cells were infected with retroviruses (pCLBCX) encoding for either GFP-LC3 or p62-GFP.^46^

For co-immunoprecipitation experiments, cells were transfected with plasmids encoding human Snail tagged with HA (Snail-HA),^47^ a kind gift of Professor Amparo Cano (Universidad Autónoma de Madrid (UAM), Madrid, Spain), alone or together with plasmids encoding for human p62-EGFP,^48^ a kind gift of Professor Terje Johansen, (University of Tromsø, Tromsø, Norway).

## Abbreviations

EMT: epithelial-to-mesenchymal transition
MET: mesenchymal-to-epithelial transition
Alb-Cre: albumin Cre recombinase transgenic mice
ATG: autophagy-related gene
MMH: Met murine hepatocytes
SQSTM1: Sequestosome-1
TGF*β*: transforming growth factor beta
Keap1: Kelch-like ECH-associated protein 1
Nrf2: nuclear factor (erythroid-derived 2)-like 2
BECN1: beclin1
GFP: green fluorescent protein
LC3: microtubule-associated protein-1 light chain 3
apoA-I: apolipoprotein A-I
apoC3: apolipoprotein C3
HNF4*α*: hepatocyte nuclear factor 4 alpha
MMP-2: matrix metalloproteinase 2
*α*SMA: alpha smooth muscle actin
HA: hematoagglutinin
EGFP: enhanced GFP
shRNA: short hairpin RNA
HNF1: hepatocyte nuclear factor 1
UPS: ubiquitin proteasome system.
