# Supplementary material for: Autophagy regulates hepatocyte identity and epithelial-to-mesenchymal and mesenchymal-to-epithelial transitions promoting Snail degradation
Source: Cell Death Dis. 2015 Sep 10;6(9):e1880–. doi: 10.1038/cddis.2015.249 (PMC4650445; doi:10.1038/cddis.2015.249)

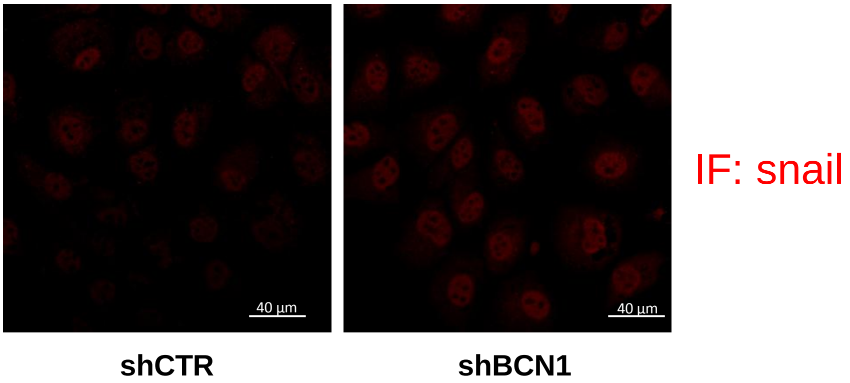
40 µm
40 µm
shCTR
shBCN1
IF: snail

Supplementary Figure 2

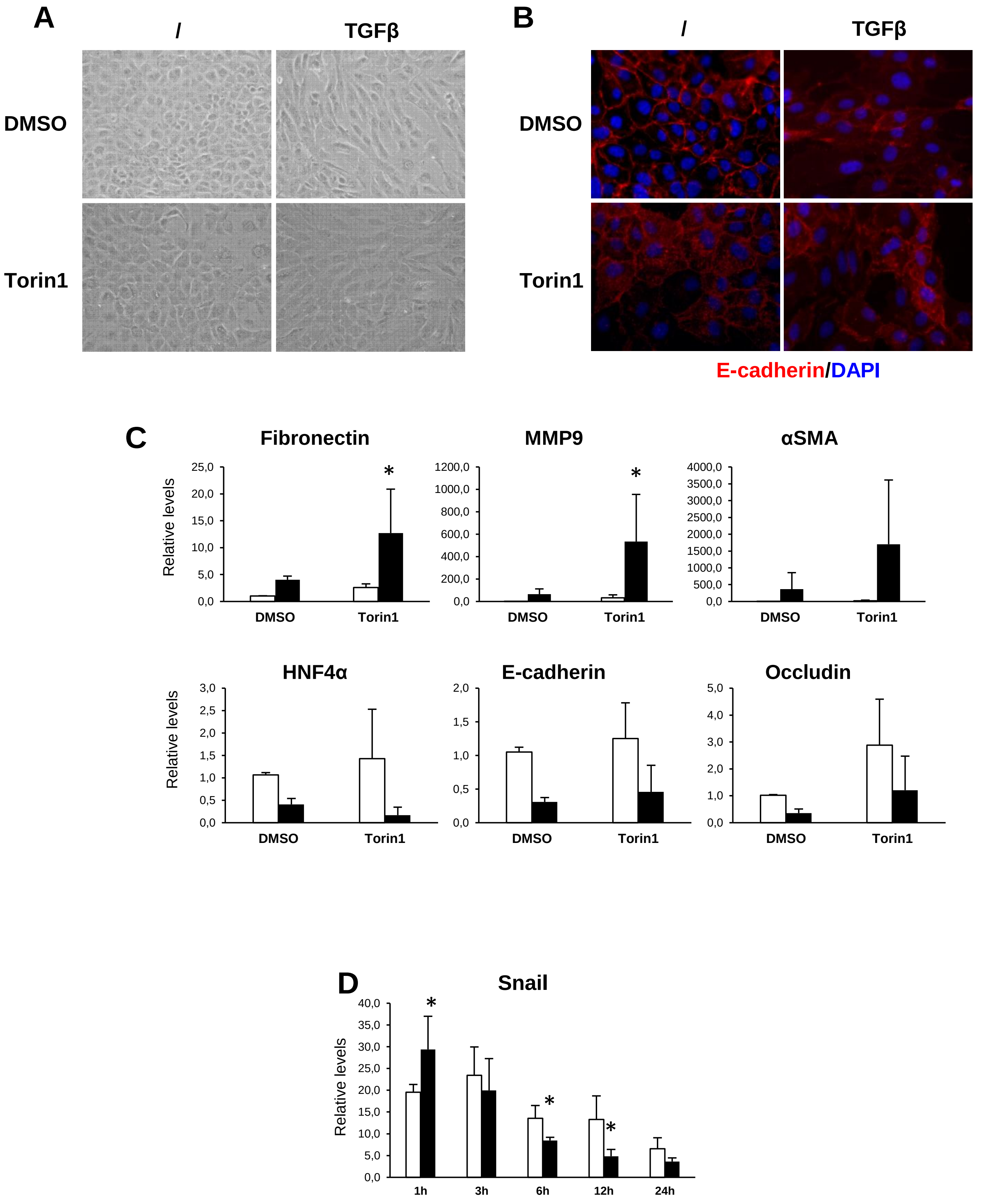

Supplementary Figure 3

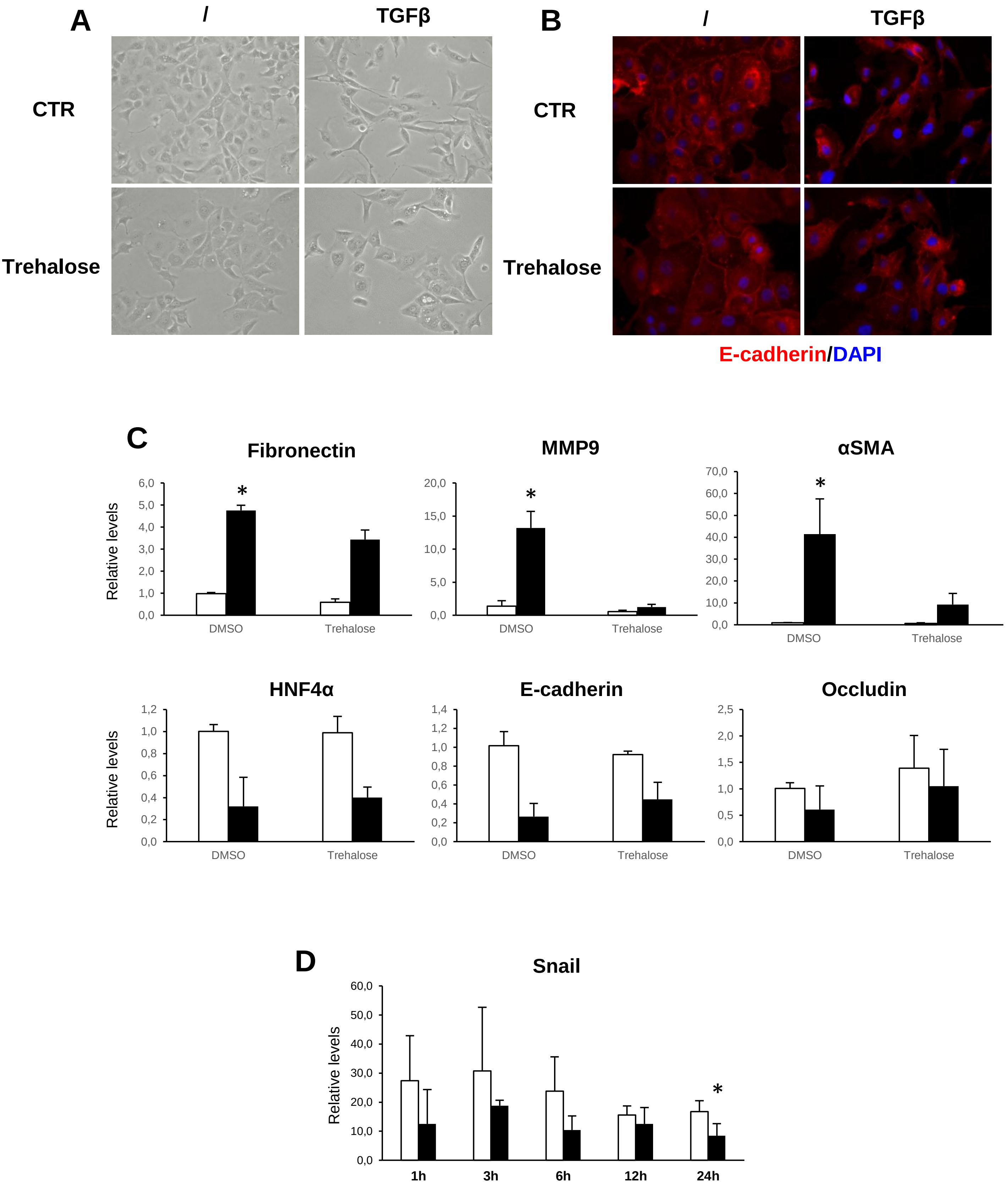

Supplementary Figure 4

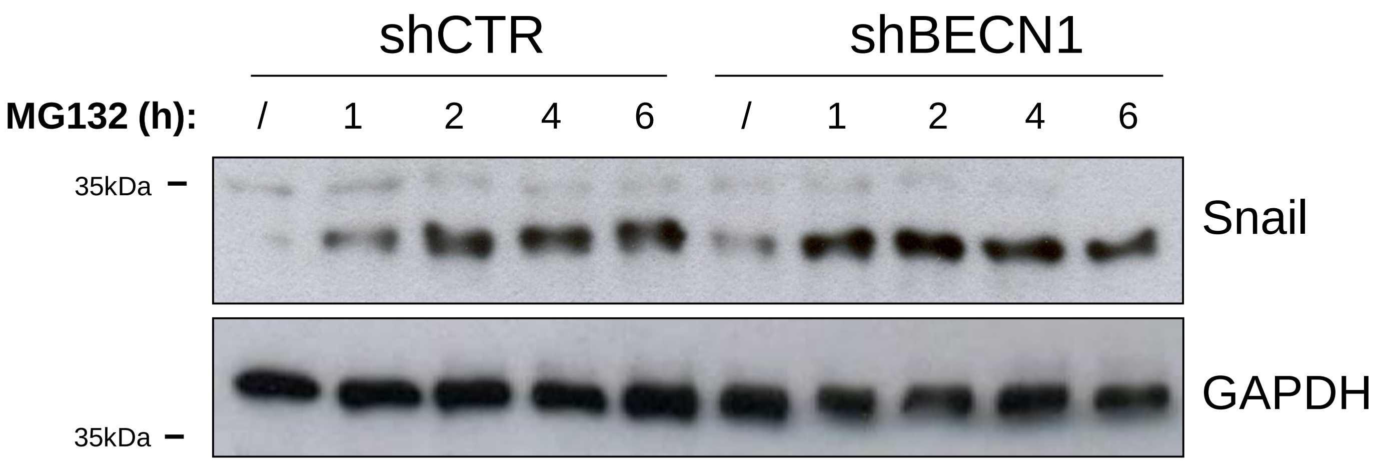

Supplementary Figure 5

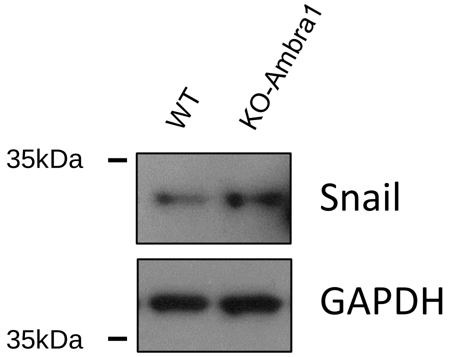

Supplement: Supplementary Figures [file cddis2015249x1.pdf]
