# Supplementary material for: Autophagy regulates hepatocyte identity and epithelial-to-mesenchymal and mesenchymal-to-epithelial transitions promoting Snail degradation
Source: Cell Death Dis. 2015 Sep 10;6(9):e1880–. doi: 10.1038/cddis.2015.249 (PMC4650445; doi:10.1038/cddis.2015.249)
Supplement: Supplementary Materials [file cddis2015249x2.doc]

**Autophagy regulates hepatocyte identity and epithelial-to-mesenchymal and mesenchymal-to-epithelial transitions promoting Snail degradation.**

Germana Grassi, Giorgia Di Caprio, Laura Santangelo, Gian Maria Fimia, Angela Maria Cozzolino, Masaaki Komatsu, Giuseppe Ippolito, Marco Tripodi, Tonino Alonzi.

Supplementary Materials:

Page 2. Supplementary Figure Legends

Page 4. Supplementary Materials and Methods

Page 10. Supplementary References

**Supplementary Figure Legends**

**Supplementary Figure 1.** **Inhibition of autophagy leads to increased levels of snail in hepatocytes**. Immunofluorescence analysis of snail in either autophagy-proficient (shCTR) autophagy-deficient (shBECN1) untreated cells.

**Supplementary Figure 2.** **Torin1-induced autophagy affects the TGFβ-mediated EMT in hepatocytes**. (A) Phase-contrast images of cells either untreated or treated with TGFβ (2ng/mL) for 24 hours in presence of Torin1 (1μM) or DMSO (0.1%v/v) as control. (B) Immunofluorescence analysis of the polarization marker E-cadherin of cells treated as in A. The blue is a DAPI nuclear counterstaining. (C-D) Levels of mRNAs of the indicated genes were measured by qPCR in DMSO- or Torin1-treated cells, as indicated, either untreated (white columns) or treated with TGFβ (2ng/mL) (black columns) for 24 hours (C) or for different hours as indicated (D). The values calculated by ΔΔCT method are relative to L34 mRNA levels. Data are expressed as mean +/- SD of three independent experiments. (*)p<0,05; p values were calculated by Mann-Whitney U test.

**Supplementary Figure 3.** **Trehalose-induced autophagy affects the TGFβ-mediated EMT in hepatocytes**. (A) Phase-contrast images of cells either untreated or treated with TGFβ (2ng/mL) for 24 hours in presence or absence of Trehalose (100mM). (B) Immunofluorescence analysis of the polarization marker E-cadherin of cells treated as in A. The blue is a DAPI nuclear counterstaining. (C-D) Levels of mRNAs of the indicated genes were measured by qPCR in untreated (CTR) or Trehalose-treated cells, as indicated, either untreated (white columns) or treated with TGFβ (2ng/mL) (black columns) for 24 hours (C) or for different hours as indicated (D). The values calculated by ΔΔCT method are relative to L34 mRNA levels. Data are expressed as mean +/- SD of three independent experiments. (*)p<0,05; p values were calculated by Mann-Whitney U test.

**Supplementary Figure 4. The ubiquitin-proteasome system (UPS) and the autophagy-lysosome pathway degrade Snail protein in hepatocytes.** Immunoblotting analysis for Snail of autophagy-proficient (shCTR) or autophagy-deficient (shBECN1) cells, either untreated or treated with the proteasome inhibitor MG132 (10μM) for the indicated times. GAPDH was used for protein loading control.

**Supplementary Figure 5. Inhibition of autophagy leads to increased levels of snail in mouse embryonic fibroblast (MEF)**.

Immunoblotting analysis for Snail of autophagy-proficient (wild type; WT) or autophagy-deficient (KO-Ambra1) untreated MEF. GAPDH was used for protein loading control.

**Supplementary Materials and Methods.**

**Cells**

The nontumorigenic immortalized MMH (Met-Murine Hepatocytes) cell lines were obtained from liver explants of cyto-Met transgenic mice at various stages of development as previously described (1). For this work a differentiated epithelial clone (MMH-D3) derived from the liver of a 3 days old mouse was used. MMH cell lines represent a well established model for the study of different aspects of hepatocyte physiopathology, such as differentiation (2-4), liver zonation, haematopoietic support (5-7), cholesterol metabolism (8), retinol binding protein regulated secretions (9,10), viral replication of HAV (11), HBV (12,13) and HCV (14), circadian clock regulation (15) and tumorigenesis (16)

The murine embryonic fibroblasts (MEF) primary cells were prepared from E13.5 embryos either wild type (WT) or with an insertion of the gene-trap vector pGT1.8geo within the Ambra1 gene (KO-Ambra1) as already described and cultured in DMEM supplemented with 20% FCS (17).

**Antibodies**

The primary antibodies used in this study were mouse anti-Snail antibody (Cell Signaling Technology, Inc, Danvers, MA for Western blot analysis), rabbit anti-Snail antibody (AbCam, Cambridge, MA for immunofluorescence analysis), rabbit anti-p62 (MBL International, Woburn, MA), rabbit anti-LC3 (Cell Signaling Technology), goat anti-HNF4α (Santa Cruz Biotechnology, Santa Cruz, CA), rabbit anti-Beclin1 (Cell Signaling Technology), rabbit anti-Twist (Santa Cruz Biotechnology, Santa Cruz, CA), mouse anti-Smooth Muscle Actin (α-SMA, Sigma-Aldrich, St Louis, MO), mouse anti-E-cadherin (BD Biosciences), rabbit anti-Slug (Cell Signaling Technology), rabbit anti-Occludin (Santa Cruz Biotechnology, Santa Cruz, CA), rabbit anti-Claudin2 (Santa Cruz Biotechnology, Santa Cruz, CA), rabbit anti-HNF1(Novus Biologicals, USA),

mouse anti-Glyceraldehyde-3-phosphate dehydrogenase (GAPDH; Calbiochem, Merck, Darmstadt, Germany), mouse anti-α-Tubulin (Santa Cruz Biotechnology), rabbit anti-Vimentin (Cell Signaling Technology).

**Immunoblotting analysis**

Liver extracts proteins (10 μg) or whole cell extracts (10 μg or 30 μg as indicated) were separated on SDS-PAGE 10% or 13,5% gels and electroblotted onto nitrocellulose (Protran; Schleicher & Schuell, Dassel, Germany) or polyvinylidene difluoride (Millipore, Billerica, MA) membranes. Blots were incubated with primary antibodies in 5% non-fat dry milk in PBS-T buffer (1X PBS, 0,1% Tween20). Detection was achieved using horseradish peroxidase-conjugate secondary antibody (Jackson ImmunoResearch Laboratories, West Grove, PA) and visualized with ECL plus (GE Healthcare Life Sciences, Little Chalfont, United Kingdom) using ECL-Hyperfilm (GE Healthcare).

**Immunoprecipitation**

For immunoprecipitation analyses cells were lysed in a buffer containing: 20 mM Tris-HCl (pH 8.0), 150 mM NaCl, 5 mM EDTA pH 7.5, 0.5% NP-40, 0.2 mM PMSF, 1mM Sodium fluoride, 1mM Sodium orthovanadate (all from Sigma-Aldrich)

One milligram of lysates was used as starting material for immunoprecipitation. Protein extracts were incubated over night at 4°C with either anti-p62 or rabbit total IgGs antibodies and immunocomplexes recovered using 25μL Protein G/Protein A Sepharose (GE Healthcare). In experiments with overexpressed tagged Snail-HA, protein extracts were incubated with 25μL anti-HA-conjugated agarose beads (Sigma-Aldrich) for 2 hours.

Immunoprecipitated complexes were analyzed by immunoblotting as described above.

**Retroviruses generation and infection**

Fifteen micrograms of pCLBCX-GFP-LC3, pCLBCX-p62-GFP or pshBECN1 were cotransfected with 5 μg of an expression plasmid for the vesicular stomatitis virus G protein into 293gp/bsr cell line by using the calcium phosphate method. Forty-eight hours later, the supernatant containing the retroviral particles was recovered and supplemented with polybrene (4 μg/mL). Hepatocytes were infected by incubation with supernatants containing retroviruses for 8 h.

**RNA interference**

RNA interference was performed using the following oligonucleotides: Beclin1 (ID: SASI_Mm01_00048143; Sigma-Aldrich), p62 (ID: SASI_Mm01_00079384 and SASI_Mm01_00079386; Sigma-Aldrich) and ATG7 (ID: MSS232487 Life Technologies). 2x10^5^ cells/well were transfected with 30 pmol siRNA in 6 well plates using Lipofectamine RNAiMAX (Life Technologies) following the supplier’s instructions.

For stable downregulation of Beclin1 (BECN1) we generated a retroviral vector (pshBECN1) cloning into BglII-HindIII sites of pSUPER.retro.puro (OligoEngine, Seattle, WA) the following oligonucleotides:

Forward 5’-GATCCCCCAAGTTTGACCATGCAATGTTCAAGAGACATTGCATGGTCAAA

CTTGTTTTTA-3’;

Reverse 5’-AGCTTAAAAACAAGTTTGACCATGCAATGTCTCTTGAACATTGCATGGTC

AAACTTGGGG-3’.

Cells with stable repression of Beclin1 were generated by infection with shBECN1 retroviruses. Selection of shBECN1 expressing cells was carried out using 2 μg/mL of puromycin.

Genes silencing was verified by immunoblotting or real time PCR analysis 48 hours after transfection.

**Immunofluorescence and confocal analysis**

For indirect immunofluorescence analyses, cells were grown on collagen I-coated 35mm dishes and fixed with 4% paraformaldehyde in 1X PBS, followed by permeabilization with 0,2% Triton X-100 in 1X PBS. Primary antibodies were incubated for 1 hour at room temperature and visualized by means of Cy3- and Alexa Fluor 488-conjugated secondary antibodies (Jackson ImmunoResearch).

Cell nuclei were stained with DAPI (Calbiochem, Merck) for indirect immunofluorescence or with the fluorochrome TO-PRO 3 (Life Technologies) for confocal analysis. Images were examined with either Zeiss Axiophot microscope for indirect immunofluorescence or with Leica TCS SP2 microscope for confocal analysis.

**Real Time PCR**

RNA was prepared with Trizol reagent (Invitrogen). cDNA synthesis was generated using the reverse transcription kit (Promega) according to manufacturer recommendations. Real-time PCR reactions were performed with Rotor Gene 6000 (Qiagen) using the Sybr Green Rox Maxima (Thermo Fisher Scientific Inc., Waltham, MA) according to the manufacturer’s instructions. 2,5 μL cDNA dilution was used as template and cycling parameters were 95°C for 10 min, followed by 40 cycles of 95°C for 10s, 62°C for 10s, 72°C for 10s. Relative amounts were obtained using the 2^–ΔΔCt^ method normalized for the L34 gene expression. GAPDH was used as additional internal control to confirm significant changes. Primer sets for all amplicons were:

| **Gene (Mus musculus)** | **Primers Sequence** |
| --- | --- |
| Albumin | For 5’-ACAGACCGGAGGGCTTATCT-3’  Rev 5’-TGGTGTAGACAGGTCAGGATGT-3’ |
| ApoAI | For 5’-CCACCTGAAGACACTTGGCG-3’  Rev 5’-TTGTCGATCACACTCTGGGCT-3’ |
| ApoC3 | For 5’-TACAGGGCTACATGGAACAAGCCT-3’  Rev 5’-AGAATCCCAGAAGCCGGTGAACTT-3’ |
| αSMA | For 5’-ACCCAGATTATGTTTGAGACC-3’  Rev 5’-CAGAGTCCAGCACAATACC-3’ |
| ATG7 | For 5’-CCTGCACAACACCAACACAC-3’  Rev 5’-CACCTGACTTTATGGCTTCCC-3’ |
| Beclin1 | For 5’-GGCCAATAAGATGGGTCTGA-3’  Rev 5’-GCTGCACACAGTCCAGAAAA-3’ |
| Claudin (Cldn1) | For 5’-ATTTGTTTCCCTGAGTGGCTGTGC-3’  Rev 5’-AGAGGAGAAGCACAGTTTGCAGGA-3’ |
| Col1A1 | For 5’-TAGACATGTTCAGCTTTGTGG-3’  Rev 5’-CTTAGGCCATTGTGTATGCAG-3’ |
| E-cadherin (Cdh1) | For 5’-CTACTGTTTCTACGGAGGAG-3’  Rev 5’-CTCAAATCAAAGTCCTGGTC-3’ |
| Fibronectin (Fn1) | For 5’-AGACCATACCTGCCGAATGTAG-3’  Rev 5’-GAGAGCTTCCTGTCCTGTAGAG-3’ |
| GAPDH | For 5’-GGCAAATTCAACGGCACAGT-3’  Rev 5’-GGCCTCACCCCATTTGATGT-3’ |
| HNF4α | For 5’- ATCTTCTTTGATCCAGATGCCA-3’  Rev 5’-GTTGATGTAATCCTCCAGGC-3’ |
| L34 | For 5’-GGTTGGGAAAGCACCTAAA-3’  Rev 5’-GACGTGCTTCTGTGTCTTAG-3’ |
| MMP2 | For 5’-CTAAGCTCATCGCAGACTCCTGGAATG-3’  Rev 5’-GGTTCTCCAGCTTCAGGTAATAAGCAC-3’ |
| MMP9 | For 5’-CTTGAAGTCTCAGAAGGTGG-3’  Rev 5’-GGCTTTGTCTTGGTACTGG-3’ |
| Occludin | For 5’-AGCAGCCCTCAGGTGACTGTTATT-3’  Rev 5’-ACGACGTTAACTCCTGAACCAGCA-3’ |
| PGC1 | For 5’-TGCAGCCAAGACTCTGTATG-3’  Rev 5’-CATCAAGTTCAGAAAGGTCAAG -3’ |
| Snail (Snai1) | For 5’-GATGGAGTGCCTTTGTACC-3’  Rev 5’-CAGTGGGTTGGCTTTAGTT-3’ |
| TIMP1 | For 5’-ATCCTCTTGTTGCTATCACTG-3’  Rev 5’-GGAACCCATGAATTTAGCCC-3’ |
| TTR | For 5’-GTCCTCTGATGGTCAAAGTC-3’  Rev 5’-CTCCTTCTACAAACTTCTCATCTG-3’ |
| Vimentin | For 5’-CAAGCAGGAGTCAAACGAG-3’  Rev 5’-CTTCCATCTCACGCATCTG-3’ |

**Supplementary References**

1. Amicone L, Spagnoli FM, Spath G, Giordano S, Tommasini C, Bernardini S, et al. Transgenic expression in the liver of truncated Met blocks apoptosis and permits immortalization of hepatocytes. EMBO J. 1997; 16(3): 495-503.

2. Mancone C, Conti B, Amicone L, Bordoni V, Cicchini C, Calvo L, et al. Proteomic analysis reveals a major role for contact inhibition in the terminal differentiation of hepatocytes. J. Hepatol. 2010; 52(2): 234-243.

3. Conigliaro A, Amicone L, Costa V, De Santis Puzzonia M, Mancone C, Sacchetti B, et al. Evidence for a common progenitor of epithelial and mesenchymal components of the liver. Cell Death Differ. 2013; 20(8): 1116-1123.

4. Garibaldi F, Cicchini C, Conigliaro A, Santangelo L, Cozzolino AM, Grassi G, et al. An epistatic mini-circuitry between the transcription factors Snail and HNF4alpha controls liver stem cell and hepatocyte features exhorting opposite regulation on stemness-inhibiting microRNAs. Cell Death Differ. 2012; 19(6): 937-946.

5. Aiuti A, Cicchini C, Bernardini S, Fedele G, Amicone L, Fantoni A, et al. Hematopoietic support and cytokine expression of murine-stable hepatocyte cell lines (MMH). Hepatology 1998; 28(6): 1645-1654.

6. Bordoni V, Alonzi T, Zanetta L, Khouri D, Conti A, Corazzari M, et al. Hepatocyte-conditioned medium sustains endothelial differentiation of human hematopoietic-endothelial progenitors. Hepatology 2007; 45(5): 1218-1228.

7. Pelosi E, Castelli G, Martin-Padura I, Bordoni V, Santoro S, Conigliaro A, et al. Human haemato-endothelial precursors: cord blood CD34+ cells produce haemogenic endothelium. PLoS One 2012; 7(12): e51109.

8. Napolitano M, Rivabene R, Avella M, Amicone L, Tripodi M, Botham KM, et al. Oxidation affects the regulation of hepatic lipid synthesis by chylomicron remnants. Free Radic. Biol. Med. 2001; 30(5): 506-515.

9. Bellovino D, Lanyau Y, Garaguso I, Amicone L, Cavallari C, Tripodi M, et al. MMH cells: An in vitro model for the study of retinol-binding protein secretion regulated by retinol. J. Cell. Physiol. 1999; 181(1): 24-32.

10. Gaetani S, Bellovino D, Apreda M, Devirgiliis C. Hepatic synthesis, maturation and complex formation between retinol-binding protein and transthyretin. Clin. Chem. Lab. Med. 2002; 40(12): 1211-1220.

11. Feigelstock DA, Thompson P, Kaplan GG. Growth of hepatitis A virus in a mouse liver cell line. J. Virol. 2005; 79(5): 2950-2955.

12. Pasquetto V, Wieland SF, Uprichard SL, Tripodi M, Chisari FV. Cytokine-sensitive replication of hepatitis B virus in immortalized mouse hepatocyte cultures. J. Virol. 2002; 76(11): 5646-5653.

13. Robek MD, Boyd BS, Wieland SF, Chisari FV. Signal transduction pathways that inhibit hepatitis B virus replication. Proc. Natl. Acad. Sci. U. S. A. 2004; 101(6): 1743-1747.

14. Uprichard SL, Chung J, Chisari FV, Wakita T. Replication of a hepatitis C virus replicon clone in mouse cells. Virol. J. 2006; 3: 89.

15. Atwood A, DeConde R, Wang SS, Mockler TC, Sabir JS, Ideker T, et al. Cell-autonomous circadian clock of hepatocytes drives rhythms in transcription and polyamine synthesis. Proc. Natl. Acad. Sci. U. S. A. 2011; 108(45): 18560-18565.

16. Gotzmann J, Huber H, Thallinger C, Wolschek M, Jansen B, Schulte-Hermann R, et al. Hepatocytes convert to a fibroblastoid phenotype through the cooperation of TGF-beta1 and Ha-Ras: steps towards invasiveness. J. Cell. Sci. 2002; 115(Pt 6): 1189-1202.

17. Antonioli M, Albiero F, Nazio F, Vescovo T, Perdomo AB, Corazzari M, et al. AMBRA1 interplay with cullin E3 ubiquitin ligases regulates autophagy dynamics. Dev. Cell. 2014; 31(6): 734-746.
